# Intra-grade variability in educational and psychosocial competencies of school going adolescent girls, in the coastal region of Kenya: implications for school based interventions

**DOI:** 10.1186/s12889-020-09097-3

**Published:** 2020-07-13

**Authors:** Beth Kangwana, Eunice Muthengi, Karen Austrian

**Affiliations:** Population Council, Kenya, Avenue 5, 3rd Floor Rose Avenue, P.O. Box 17643–00500, Nairobi, Kenya

**Keywords:** Adolescent girls, Psychosocial indicators, Education, Empowerment, Competencies

## Abstract

**Background:**

The onset of puberty and menarche is a potentially vulnerable time for girls. Educational and psychosocial competencies are regarded as essential tools that empower them to successfully navigate the adolescent years. The aim of this study is to evaluate to what extent school going girls are equipped with these key competencies, and how they vary across a given grade cohort.

**Methods:**

Data was collected in Kilifi County, Kenya, from 140 public primary schools from grade 7, across three sub-counties. Bivariate and multivariate analyses were carried out to compare competency outcomes by age groups comprising 10–14 years and 15 year and above. Generalized estimating equations with robust standard errors was used where outcomes were measured as binary outcomes, and linear regression for continuous outcomes. Clustering was factored in at the school level and stratification at the subcounty level. Wilcoxon Rank sum test incorporating clustering effects was used where continuous outcomes were not normally distributed.

**Results:**

A total of 3489 adolescent girls were interviewed with a mean age of 14 years (SD:1.5; min:10, max:21). Compared to the lower age group, girls in the higher age group were less likely to have ambitions of furthering their education beyond secondary school (odds ratio (OR):0.63 (95%CI:0.53, 0.74)), more likely to report not feeling confident enough to answer questions in class (OR:1.18 (95%CI:1.02, 1.36) and scored lower on their cognitive, math and literacy tests. They also displayed less positive gender norms (coefficient (coeff):-0.091 (95%CI:-0.16, − 0.022)) and were more likely to agree with intimate-partner violence in marriage (coeff:1.17 (95%CI:1.00, 1.37)). They however scored higher on the decision-making scale (coeff:0.36 (95%CI:0.13, 0.60)) and were more likely to be able to spontaneously name a method of modern contraception (OR:1.56 (95%CI:1.36, 1.80)).

**Conclusion:**

Large variability in age exits within a grade. Compared to older girls, younger girls were more likely to perform better on their educational and social competencies. In countries with large age ranges per grade, identifying the presence of educational and psychosocial competency variabilities will allow informed decisions to be made on how school-based interventions should be adapted to address the varying needs within a grade.

**Trial registration:**

ISRCTN10894523, date of registration: 22/08/2017. Retrospectively registered.

## Background

The onset of puberty and menarche is a period in life marked with significant developmental and social changes [1]. During this time, biological maturity precedes psychosocial maturity, and younger adolescent girls become vulnerable to external pressures, such as to initiate sexual activity, and at times sexual coercion from boys and men [2]. Compounded to this are certain expectations from themselves and their families which may include early marriage or the need to perform well in primary school in order to transition to secondary school [2]. According to several qualitative studies in Africa, such pressures are exacerbated by girls’ lack of understanding of their bodies, their rights, implications of their decisions, and by their inability to manage puberty and adolescence safely and comfortably [3–8].

According to Lloyd (2013) [9], educational, and psychosocial competencies, which encompass social, personal and economic competencies, are regarded as essential tools that empower girls to successfully navigate the adolescent years. Educational competencies, including reading, writing and problem solving are correlated with increased knowledge and greater skills for remunerative work [10]. Personal competencies, such as sexual and reproductive health (SRH) knowledge, have shown to delay or decrease sexual behavior and encourage the use of family planning methods, in turn delaying pregnancy and engagement in sexual activities [11]. Personal competencies which includes self-efficacy, have been demonstrated to act as psychological mediators of health and academic accomplishment, as well as having an indirect impact on positive social behavior [12, 13]. Social competencies including gender consciousness, negotiating skills and leadership skills have shown to significantly lower the rate of sexually transmitted infections (STIs) or unintended pregnancy [14]. Finally, economic competencies such as financial literacy, resource management and entrepreneurship have shown to be correlated to girls being able to secure income and become self-sufficient [15].

Adolescence can be separated into two groups, early adolescence who are 10–14 year olds, and late adolescence who are 15–19 years; young adults fall into the age category of 20–24 years [1]. In early adolescence physical changes generally commence and the frontal lobe, the part of the brain that governs reasoning and decision making starts to develop; adolescents at this age tend to be more impulsive and uncritical in their thinking [1]. By late adolescent the capacity for analytical and reflective thought is greatly enhanced and adolescents begin to gain more clarity and confidence in their own identity and opinions. These differences in mental and physical developments across these age groups are important to consider when designing and implementing interventions that promote positive health behavior [1].

The aim of this study is to evaluate to what extent school going girls, in a relatively poor coastal region of Kenya, are equipped with a range of key competencies and how these competencies vary across girls in a given grade cohort. Such information will be valuable in informing the process of designing and implementing programs in order to enhance empowerment of adolescent girls, and therefore improve potential longer-term outcomes such as school retention, reduced unwanted sex, delayed first sex and delayed pregnancy.

## Methods

### Study site

The study was conducted in Kilifi County in the coastal region of Kenya. This area was selected because of its poor performance indicators in relation to education and reproductive health. Within this county, an estimated 40% of primary school children transition to secondary school, compared to the national rate of 72%; the net enrollment rate for secondary school boys and girls was at 26% in 2014 [16, 17]. Approximately 22% of girls begin childbearing between the ages of 15 and 19, compared to the national average of 18% [18].

### Study design

This study makes use of data from the baseline of a larger cluster randomized controlled trial designed to assess the impact of provision of sanitary pads and reproductive education in schools on increased school retention and sexual reproductive health outcomes [19]. At baseline, a cross-sectional study was carried out in 140 public primary schools, across three rural subcounties which were selected in collaboration with the Kilifi County Department of Education, Ministry of Education, Science and Technology.

**Fig. 1:**
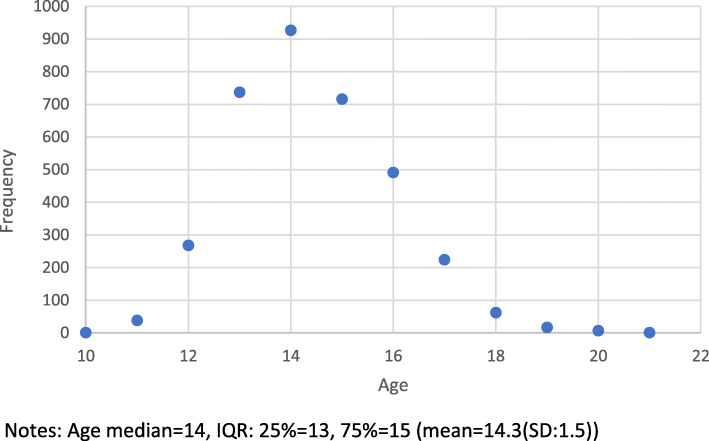
Age Range of Girls in Grade 7. Notes: Age median = 14, IQR: 25% = 13, 75% = 15 (mean = 14.3(SD:1.5))

All primary schools in the county with 25 or more girls in grade 7 were eligible for the study. This totaled to 215 schools, which were mapped using geographic positioning, and a 1000-m buffer created around each school. For schools with overlapping boundaries, one school was randomly selected. This resulted in a list of 173 schools with at least 25 girls in grade 7. A listing exercise was conducted to verify enrollment and school type in the first quarter of 2017. Based on this data, 25 schools were excluded because they did not meet the minimum criteria of 25 girls in grade 7, and an additional seven were excluded because they were determined to be boarding schools. Subsequently, one school was excluded after it was closed indefinitely by the Department of Education during data collection. In total, 140 primary schools formed the final sample. In schools with 25 girls in grade 7, all girls were included in the research sample. In schools with a larger number of girls in grade 7, 25 girls were randomly selected for the research sample and five additional girls were selected as alternates in case sample students were not attending on the day of interviews (Fig. 1).

### Data collection

Data collection took place between February and May 2017, before training and implementation of the intervention. Trained female interviewers visited villages associated with selected schools to locate the households of selected girls. To the extent possible, interviews were conducted in a private area, with visual and auditory privacy, either at the home or the school. The survey was conducted in either English or Swahili using tablet computers and programmed with Open Data Kit software. For sensitive questions regarding sexual behavior, the Audio Computer-Assisted Self Interviewing (ACASI) technique was used. With ACASI, respondents were provided with headphones through which they heard recordings of questions and response categories in their language of choice. They were then prompted to enter a response by touching a designated color for each response option. The girls’ survey included topics such as: socio-demographic characteristics, education participation and engagement, friendship networks, self-esteem, trusting relationships with adults, self-efficacy, decision-making, financial literacy, menstruation, reproductive health knowledge, gender norms, sexual behavior, gender-based violence, literacy, mathematical assessments, and cognitive testing.

### Sample size

Calculations of statistical power were used to determine the number of clusters (i.e., schools) and the number of girls needed in each cluster to observe the desired change in primary outcomes of the impact evaluation, specifically the mean days of school missed, according to differences detected in previous studies conducted in Kenya and Ghana [20, 21]. Further details on the sample size calculation can be found in the study protocol [19].

### Data analysis

#### Outcomes

The paper focused on educational and psychosocial competency outcomes, specifically personal and social psychosocial competencies. Educational competencies consisted of school participation and engagement (using questions from the Adolescent Girls Initiative-Kenya study, [22]) as well as cognitive (using a subset of Raven’s Coloured progressive matrices, [23]), literacy and mathematics skills (using excerpts from the Uwezo Kenya National Learning Assessment [24]). Personal competencies consisted of sexual and reproductive health knowledge. Knowledge on pregnancy and sexually transmitted infections were each assessed by asking 4 questions, and one point was given for each question answered correctly. General self-efficacy was measured using a 10-point scale and consisted of questions reflecting self-efficacy to 10 agree/disagree survey items, for example “You always manage to solve difficult problems if you try hard enough” [25]. Decision making was measured using a 13-point scale consisting of questions reflecting decision making to 13 often/sometimes/ rarely or never survey items, for example “My parents or guardians ask for my opinion on things”, and how often one makes certain decisions without an adult. In the analysis the response option “often” to a positive decision-making statement was counted as one point and “sometimes/ rarely or never” was counted as zero [26]. Social competencies consisted of positive gender norms which was measured using a four-point scale consisting of four questions reflecting gender-norms to four agree/ disagree survey items. Both decision making and social competency scales were source from the global early adolescent study (GEAS) and adapted to the local context [26]. Acceptability of intimate-partner violence (IPV) was measured by asking the respondent whether they agreed or disagreed on any of four different IPV scenarios presented to them and was sourced from the Kenya National Demographic Health Survey [18], for example “If a man doesn’t hit his wife, it means he doesn’t love her”. SRH experiences were reporting on having started menstruation and have ever had sex. The ACASI method was used to collect data on sexual behavior [27] and ‘ever having sex’ was defined as yes if the respondent provided an age to the following question ‘… *how old were you when you had sexual intercourse for the first time*?’. The survey instrument was designed specifically for the purposes of this study [28]. The outcomes were compared across two age categories: 10–14 year old which are also known as early adolescents, and 15 and above which combines late adolescence and young adulthood. Only 8 girls were above 19 years old therefore the older age group largely fell into the late adolescent age category of 15 to 19 years.

#### Data analysis

Descriptive analysis was conducted using STATA [version 14.1] to calculate frequencies, percentages, and means for key indicators. Some indicators were measured using scales created from a list of related variables. The internal consistency or average correlation of items was measured using Chronbach’s alpha. Bivariate analysis was carried to compare educational and psychosocial outcomes across the two age groups. Generalized estimating equations (GEE) with robust standard errors was used where psychosocial outcomes were measured as binary outcomes, and linear regression for continuous outcomes. Clustering was factored in at the school level and stratification at the subcounty level. Wilcoxon Rank sum incorporating clustering effects was used in instances where residuals from the continuous outcome data was not normally distributed. Multivariate analysis was carried out to compare educational and psychosocial outcomes across the two age groups controlling for socio-economic status, who the participant lives with, parental survival status and religion.

## Results

A total of 3489 adolescent girls in grade 7 were interviewed. More than half of respondents were between the ages of 10 and 14 years (57%) and 44% were between the ages of 15 and 21 years (see Table 1 & Fig. 1). Approximately 84% of respondents were Christian and 13% Muslim, and 3% reported another religion or no religion. About 82% of girls reported that both parents were still alive, while 13% had lost a father, 2% had lost a mother, and 1% were total orphans. Half of respondents (56%) were reported as residing with both parents at the time of the survey. A quarter of girls (26%) were residing with their mother only, 3% with their father only, and 15% with neither parent (see Table 1). Girls in the poorest households were more likely to fall into the older age group when compared to the younger age group (26% vs 15%) while those in the least poorest households were more likely to fall into the 10–14 years age group when compared to the older age group (27% vs 11%). The mean age of starting primary school was 7 and 8 years in the 10–14 and 15 plus year age groups respectively, however close to one third of girls reported not knowing what age they started primary school (not shown in tables).

**Table 1:** Characteristics of Respondents

**Variable**	**10–14 years** **n (%)**	**15 and above** **n (%)**	**Total** **n (%)**
Total	1971 (56.5)	1518 (43.5)	3489
Religion			
Christian	1675 (85.0)	1270 (83.7)	2945 (84.4)
Islam	261 (13.2)	194 (12.8)	455 (13.0)
No religion/ other	35 (1.8)	54 (3.6)	89 (2.6)
Parental Survival Status			
Only mother alive	233 (11.8)	222 (14.6)	455 (13.1)
Only father alive	36 (1.8)	41 (2.7)	77 (2.2)
Both parents alive	1631 (82.8)	1220 (80.4)	2851 (81.8)
Parents not alive	28 (1.4)	22 (1.5)	50 (1.4)
Don’t know if father alive	43 (2.2)	11 (0.7)	54 (1.6)
Who does participant live with			
Mother, not father	506 (25.7)	393 (25.9)	899 (25.8)
Father, not mother	56 (2.8)	55 (3.6)	111 (3.2)
Both mother and father	1109 (56.3)	860 (56.7)	1969 (56.4)
Neither mother nor father	300 (15.2)	210 (13.8)	510 (14.6)
Household Socio-Economic Status (n(%))			
Quintile 1 (poorest households)	303 (15.4)	395 (26.0)	698 (20.0) ^a^
Quintile 2	343 (17.4)	355 (23.4)	698 (20.0)
Quintile 3	370 (18.8)	327 (21.5)	697 (19.9)
Quintile 4	430 (21.8)	268 (17.7)	698 (20.0)
Quintile 5 (least poor households)	524 (26.6)	173 (11.4)	697 (20.0)

^a^*p* < 0.001 (Chi squared test)

In the bivariate analysis (Table 2), when girls were asked what the highest level of schooling they would like to achieve was, those in the higher age group were less likely to have ambitions of furthering their education beyond secondary school compared to the younger group (odds ratio (OR) 0.59 (95% Confidence Intervals (CI): 0.50, 0.69)). Girls were asked what was the highest level of schooling they actually expected to complete: 58 and 47% of girls expected to stop education at secondary school in the higher and lower age groups respectively (OR:0.68 (95% CI: 0.59, 0.77)), and more than 90% across both groups reported that the main reason they believed they could not go beyond secondary school was not being able to afford school fees (not shown in tables). Girls in the older age group were more likely to have repeated a grade compared to their younger counter parts (OR:4.10 (95% CI:3.50, 4.81)), more likely to report not feeling confident enough to answer questions in class (OR:1.24 (95% CI:1.07, 1.43)), and more likely to report often not understanding what is being taught in the lessons (OR:1.47 (95% CI:1.27,1,71)). Girls in the higher age group also scored lower on their cognitive (coefficient (coeff):− 1.13 (95% CI:-1.36, − 0.091)), literacy (coeff:-0.19 (95% CI:-0.24, − 0.13)) and mathematics skills (coeff:-1.26 (95% CI:-1.58, − 0.94)) tests compared to girls in the lower age group (Table 2).

**Table 2:** Competencies and Sexual and Reproductive Behavior of Girls in Grade 7 by Age Categories

**Variable**	**10–14 years**	**15 plus years**	**OR/Coeff (95% CI)** ***P*-value** **unadjusted**	**OR/Coeff (95% CI)** ***P*-value** adjusted*
**Educational competencies (n(%))**				
Highest level of school you would like to complete				
Up to secondary school	305 (15.5)	372 (24.5)	ref	ref
Beyond secondary school	1666 (84.5)	1146 (75.5)	0.59 ^b^ (0.50, 0.69) < 0.001	0.63^b^ (0.53, 0.74) < 0.001
Highest level of schooling you actually expect to complete				
Up to secondary school	922 (46.8)	874 (57.6)	ref	ref
Beyond secondary school	1049 (53.2)	644 (42.4)	0.68^b^ (0.59, 0.77) < 0.001	0.78 ^b^ (0.68, 0.89) < 0.001
Ever repeated a grade				
No	927 (47.0)	261 (17.2)	ref	ref
Yes	1044 (55.0)	1257 (82.8)	4.10 ^b^ (3.50, 4.81) < 0.001	4.12 ^b^ (3.51, 4.82) < 0.001
School engagement (n(%))				
You do not feel confident answering questions in class (agree)	640 (32.6)	564 (37.3)	1.24 ^b^ (1.07, 1.43) 0.003	1.18 ^b^ (1.02, 1.36) 0.025
You always complete your school work (agree)	1837 (93.3)	1412 (93.0)	0.96 ^b^ (0.74, 1.22) 0.732	1.03 ^b^ (0.80, 1.32) 0.829
You believe you are capable of doing well in school (agree)	1917 (97.6)	1461 (96.6)	0.70 ^b^ (0.45, 1.08) 0.109	0.70 ^b^ (0.45, 1.09) 0.119
You often do not understand the lessons (agree)	648 (32.9)	632 (41.6)	1.47 ^b^ (1.27, 1.71) < 0.001	1.37 ^b^ (1.18, 1.60) < 0.001
Educational skills (mean,(SD),n)				
Cognitive skills (score:0–15)	9.5 (3.0) 1971	8.3 (3.1) 1518	-1.13^c^ (−1.35, −0.091) < 0.001	−0.99 ^c^ (−1.19, −0.78) < 0.001
Literacy skills (score:0–4)	3.9 (0.5) 1971	3.8 (0.8) 1518	− 0.19 ^c^ (− 0.24, − 0.13) < 0.001	− 0.19 ^c^ (− 0.25, − 0.13) ^d^ < 0.001
Mathematics skills (score:0–38)	30.2 (3.5) 1971	28.9 (4.2) 1158	− 1.26 ^c^ (− 1.58, − 0.94) < 0.001	− 1.12 ^c^ (− 1.43, − 0.81) < 0.003
**Personal Competencies**				
Self-efficacy (mean,(SD),n) (scale:0–10; alpha: 0.7337)	5.3 (2.6) 1971	5.3 (2.6) 1518	− 0.015 ^c^ (− 0.19, 0.16) 0.869	0.036 ^c^ (−0.15, 0.22) 0.702
Decision-making (mean,(SD),n) (scale:0–13; alpha: 0.7790)	6.1 (3.3) 1971	6.4 (3.2) 1518	0.32 ^c^ (0.086, 0.54) 0.007	0.36 ^c^ (0.13, 0.60) 0.002
SRH Knowledge (mean,(SD),n)				
Pregnancy knowledge (score:0–4)	2.5 (0.6) 919	2.5 (0.6) 761	−0.045 ^c^ (− 0.10,0.0096) 0.105	−0.040 ^c^ (− 0.097, 0.017) 0.171
Can spontaneously name a method of modern contraception (n (%))	908 (46.1)	870 (57.3)	1.60 ^b^ (1.38, 1.83) < 0.001	1.56 ^b^ (1.36, 1.80) < 0.001
STI knowledge score (score:0–4)	0.42 (0.92) 1971	0.43 (0.91) 1518	0.0068 ^c^ (−0.031, 0.045) 0.727	0.0078 ^c^ (−0.031, 0.046) 0.687
HIV knowledge score (score:0–11)	8.0 (1.8) 1971	7.9 (1.8) 1518	−0.017 ^c^ (− 0.043, 0.0089) 0.197	−0.017 (− 0.043, 0.009) 0.192
Sexual and Reproductive Health Experiences (n(%))				
Started menstruation	1258 (67.0)	1467 (97.2)	16.79 ^b^ (12.28, 22.95) < 0.001	16.37 ^b^ (11.97, 22.39) < 0.001
Ever had sex	145 (7.4)	249 (16.4)	2.43 ^b^ (1.96, 3.02) < 0.001	2.33 ^b^ (1.85, 2.91) < 0.001
**Social competencies**				
Gender Norms (mean,(SD),n) (score:0–4; alpha: 0.6529	1.39 (1.04) 1971	1.29 (1.04) 1518	−0.11 (−0.18, − 0.04) 0.002	−0.091 (− 0.16, − 0.022) 0.010
GBV: Agrees with IPV in marriage (n(%))	1258 (63.8)	1061 (69.9)	1.26 ^b^ (1.08, 1.47) 0.003	1.17 ^b^ (1.00, 1.37) 0.050

^b^Odds ratios derived using generalized estimating equations and robust standard errors, factoring clustering at the school level and stratified by subcounty

^c^ Linear regression incorporating clustering effects or Wilcoxon Rank sum test was used in instances where continuous outcome data was not normally distributed

^d^Difference between adjusted and unadjusted numbers tended to occur after 3 decimal points

*Adjusted for socio-economic status, who the girl lives with, parent survival status and religion

When it came to personal competencies, girls in the higher age group scored higher on the decision-making scale (coeff: 0.32 (95% CI: 0.086, 0.54)); and were also more likely to be able to spontaneously name a method of modern contraception compared to those in the lower age group (OR: 1.60 (95% CI:1.38, 1.83)). No other differences were observed between the groups on the other personal competencies including self-efficacy and other sexual and reproductive health knowledge scores. Girls in the higher age group were more likely to have started menstruation (OR:16.37 (95% CI: 12.28, 22.95)) and reported to have ever had sex (OR:2.33 (95% CI:1.96, 3.02)), compared to girls in the lower age group (Table 2).

Social competencies consisted of gender norms and agreeing to IPV in marriage. Girls in the higher age group displayed less positive gender norms (coeff:- -0.11 (95% CI: − 0.18, − 0.04)), and were more likely to agree with inter-partner violence in marriage (OR:1.26 (95% CI:1.08. 1.47)), compared to girls in the lower age group (Table 2).

In the multivariate analysis (Table 2), after controlling for socio-economic status, religion, who the girl lives with and parental survival status, meaningful differences observed between the two age groups remained the same to those observed in the bivariate analysis.

## Discussion

The average age for grade 7 in Kenya should be 12 years old if one starts grade 1 at age 6 and has no breaks in schooling. Overall, the findings display a wide age distribution of girls within grade 7, ranging from 10 to 21 years. Even within one grade, meaningful differences in a range of competencies exist between girls who fall into early (10–14 years) and late (15–19 years) adolescence, especially when it comes to educational and social competencies, as well as sexual and reproductive health experiences. These wide differences in age range within a grade have been demonstrated in other studies in sub Saharan Africa [29, 30].

Early adolescent girls displayed significantly higher educational competencies over a range of outcomes compared to their older counterparts. Younger girls did not only perform better in the cognitive, literacy and math skills tests, but they also had greater aspirations of completing education beyond secondary school, felt more confident answering questions in class and understood what was being taught in the lessons more. Late adolescent girls were more likely to have repeated a grade, which partly explains why they are in a lower grade than they should be for their age, and were more likely to expect that their level of schooling would end at high school. Although older girls were much more likely to come from lower socio-economic households, almost half of all girls felt that their inability to pay for school fees would hold them back from being able to continue education beyond secondary school. Findings from this study suggest that interventions aiming to improve girls’ schooling outcomes in this setting should be targeted at girls who are older than the expected average grade age, and need not just to focus on persuading girls about the importance of pursuing education, but addressing the barriers that may prevent them from reaching their aspirations or even wanting to have greater educational aspirations. When it came to personal skills, older girls were significantly more competent in decision making than younger girls, and this could likely be a reflection of them being older and more mature [31] . There was no difference observed across the groups when it came to self-efficacy.

Girls in the late adolescent age group possessed poorer social skills compared to early adolescents, displaying less positive gender norms and being more likely to support IPV, although the indicators on social competencies observed across both age groups were generally low. The findings suggest that there is substantial room for improvement on the girls’ social and personal competencies across both age groups and that the factors influencing the development of social and personal competencies are different from those that may delay girls schooling.

Similarly, the limited SRH knowledge across both age groups points to the need for a more comprehensive curriculum focusing on these issues. Girls in the older age groups were only more knowledgeable than their younger counterparts on being able to mention a modern contraception; not only was there no difference in knowledge on pregnancy and STIs between the two groups but also knowledge on these topics across the two groups was generally low. The relatively low scores on SRH knowledge is of concern especially in girls in the older age group as they reported being more than twice more likely to have ever engaged in sex compared to their younger counterparts. There is therefore an urgent need to target sexual and reproductive health knowledge especially at the older group of girls.

This study had several strengths: The large sample size of over 3000 girls across 140 schools, and the high response rate reduced the potential of selection bias and ensured that the results were a representative description of girls in Grade 7 in those sub-counties. In addition, the data collection tools were comprehensive, capturing information on adolescent girls’ education, and social and personal competencies, therefore providing a multi-faceted picture which can be correlated to a general level of empowerment [9]. Limitations of the study included reporting bias: although girls were allowed to answer sensitive questions in private, there was the possibility of some girls over or under reporting on certain questions in order to protect their reputation. Girls were asked ‘*how old were you when you had sexual intercourse*’ to identify those who had ever had sex. It is possible that girls may have not clearly understood what the definition of sex is. The median age of reporting initiation of sex was 12 and 14 years, in the 10–14 year age group and 15 and above age group, respectively. Age of first sex is therefore likely capturing data on early sexual initiators and is not reflective of the whole sample. It is likely that age of first sex will continue to rise as the cohort grows older and more girls experience sex for the first time. Furthermore, the data in this paper are cross-sectional and therefore only associations can be determined. Lastly, in terms of generalizability, Kilifi County was selected as the study site because of its poor educational and SRH outcomes. It will therefore be difficult to generalize these findings to other regions of the country, however the data does provide insight into what may be seen in regions both within and outside of the country with similar socio-economic profiles.

## Conclusion

This study highlights that even within a grade there is large variability in age and this is associated with significant differences in competencies across the different age groups. It is important to identify the presence of these variabilities and use this information to inform how interventions need to be adapted to the address the different needs within the same grade. This is likely to enhance the impact of school-based interventions targeted at empowering girls and promoting positive outcomes such as school retention, reduced unwanted sex, delayed first sex and delayed pregnancy.

## Data Availability

The datasets analysed in this study are available from the corresponding author on reasonable request.

## Abbreviations

ACASI: Audio Computer-Assisted Self Interviewing
STI: Sexually Transmitted Infections
IPV: Intimate-Partner Violence
SRH: Sexual and Reproductive Health
GEAS: Global Early Adolescent Study
